# Multiplexed Quantum Dot Labeling of Activated c-Met Signaling in Castration-Resistant Human Prostate Cancer

**DOI:** 10.1371/journal.pone.0028670

**Published:** 2011-12-21

**Authors:** Peizhen Hu, Gina C.-Y. Chu, Guodong Zhu, Hua Yang, Daniel Luthringer, Gail Prins, Fouad Habib, Yuzhuo Wang, Ruoxiang Wang, Leland W. K. Chung, Haiyen E. Zhau

**Affiliations:** 1 Uro-Oncology Research Program, Department of Medicine, Cedars-Sinai Medical Center, Los Angeles, California, United States of America; 2 Department of Pathology, Xijing Hospital, Fourth Military University, Xi'an, China; 3 Department of Urology, The First Affiliated Hospital of Medical School, Xi'an Jiaotong University, Xi'an, China; 4 Department of Pathology, Jilin University, Changchun, China; 5 Department of Pathology, Cedars-Sinai Medical Center, Los Angeles, California, United States of America; 6 Department of Physiology, University of Illinois at Chicago, Chicago, Illinois, United States of America; 7 Department of Surgery, The University of Edinburgh, Edinburgh, Scotland; 8 Department of Experimental Therapeutics, BC Cancer, Vancouver, British Columbia, Canada; The University of Texas M.D Anderson Cancer Center, United States of America

## Abstract

The potential application of multiplexed quantum dot labeling (MQDL) for cancer detection and prognosis and monitoring therapeutic responses has attracted the interests of bioengineers, pathologists and cancer biologists. Many published studies claim that MQDL is effective for cancer biomarker detection and useful in cancer diagnosis and prognosis, these studies have not been standardized against quantitative biochemical and molecular determinations. In the present study, we used a molecularly characterized human prostate cancer cell model exhibiting activated c-Met signaling with epithelial to mesenchymal transition (EMT) and lethal metastatic progression to bone and soft tissues as the gold standard, and compared the c-Met cell signaling network in this model, in clinical human prostate cancer tissue specimens and in a castration-resistant human prostate cancer xenograft model. We observed c-Met signaling network activation, manifested by increased phosphorylated c-Met in all three. The downstream survival signaling network was mediated by NF-κB and Mcl-1 and EMT was driven by receptor activator of NF-κB ligand (RANKL), at the single cell level in clinical prostate cancer specimens and the xenograft model. Results were confirmed by real-time RT-PCR and western blots in a human prostate cancer cell model. MQDL is a powerful tool for assessing biomarker expression and it offers molecular insights into cancer progression at both the cell and tissue level with high degree of sensitivity.

## Introduction

Semi-conductor quantum dots (QDs) fluorescent nanoparticles have been recognized as one of the great recent advances for our ability to detect relevant biomarkers expressed by cells, tissues and sera [1], [2], [3], [4], [5]. The unique optical and electronic properties of QDs include their narrow and symmetrical emission bands, size- and material-tunable light emission, high surface to volume ratio, photostability, signal brightness and sensitivity, and simultaneous excitation of multiple fluorescence colors making it possible to detect multiple targets simultaneously at the single cell level [3], [6]. Quantum dot labeling, QDL, is superior to conventional organic dyes for cell and tissue staining, especially since the latter yield broad bandwidth with overlapping signal emissions and are highly susceptible to photobleaching. Multiplexing biomarkers with different colors provides significant advantages over traditional organic or fluorescent dyes for the detection and analysis of dynamic changes in proteins and nucleic acids in cells or tissues under pathophysiologic conditions. QDL has been applied successfully to detect the levels of expression of genes *in situ* associated with important biologic processes such as epithelial to mesenchymal transition [6] in cancer metastasis [7], protein biomarkers in the blood, the presence of nucleic acids, microRNA and DNA methylation in sera or tissue extracts with samples barcoded for rapid processing by automated protocols [8], [9], [10]. In the present study, we employed multiplexed quantum dot labeling (MQDL) to detect the activated c-Met-mediated cell signaling pathway leading to EMT, cancer growth and bone and soft tissue metastasis in a novel prostate cancer metastasis model [11], [12], [13]. We confirmed the levels of gene expression assessed by MQDL with gene expression as determined by RT-PCR and western blots. We then applied the MQDL protocol to determine if the c-Met cell signaling pathway and EMT are activated in a castration-resistant prostate cancer (CRPC) animal model and in clinical prostate cancer specimens obtained from patients with high Gleason scores and bone metastasis. We observed that activation of c-Met is closely linked to EMT in cancer cells and their subsequent increased migration, invasion and metastasis [14], [15], [16]. c-Met signal activation in human prostate cancer has important clinical implications: 1) c-Met downstream signaling drives EMT and cancer cell migration, invasion, metastasis and survival in several human solid tumor models including prostate cancer [15], [17], [18], [19]. 2) Since targeting c-Met and VEGFR2 downstream signaling with a synthetic multiple tyrosine kinase inhibitor, cabozantinib (XL-184), resulted in remarkable resolution of bone and soft tissue metastases in a large number of patients with solid tumors including CRPC patients [20], [21], it would be important to show if these cell signaling pathways might be activated in clinical specimens and in relevant tumor xenograft models. We used a human prostate cancer cell line to demonstrate that c-Met activation confers EMT and prostate cancer bone and soft tissue metastases and investigated in depth the c-Met signaling activation by molecular analyses with results confirmed by MQDL. We then employed MQDL to evaluate c-Met signaling activation in clinical prostate cancer tissue specimens and correlated the results with a castration-resistant human prostate cancer xenograft model. We showed that MQDL, coupled with Vectra Image Analysis, enhances the quantitative profiling capability of individual biomarkers at the single cell level. A series of biomarkers associated with EMT, such as decreased expression of EpCAM, and increased expression of N-cadherin, vimentin and RANKL, and c-Met signal activation, including VEGF, neuropilin 1, p-c-Met and phospho(p)-NF-κB p65 [15], [22], were analyzed. The results of these studies showed a remarkable parallelism of EMT and c-Met activation between the prostate cancer cell model, the CRPC xenograft model and clinical prostate cancer specimens. The methodologies established in the present study could be of significant value in the near future to characterize other activated signaling pathways in clinical specimens, interrogate molecular mechanisms underlying cancer progression, identify druggable targets, and follow up the clinical response of patients to therapeutic intervention.

## Materials and Methods

### Cell culture and cell transfection

The LNCaP cell line, kindly provided by late Dr. Gary Miller of the University of Colorado (Denver, CO), was cultured in RPMI 1640 medium (Invitrogen) supplemented with 5% fetal bovine serum (Atlanta Biologicals, Lowrenceville, GA), penicillin (100 µg/ml) and tetracycline (100 u/ml) at 37°C in humidified atmosphere with 5% CO_2_. The establishment of clones overexpressing RANKL protein was reported previously [23]. Briefly, a full-length cDNA encoding human RANKL from OriGene (Rockville, MD) was cloned unidirectionally to p3×FLAG- myc-CMV-25 (Sigma-Aldrich, St. Louis, MO) at the NotI and XbaI restriction enzyme sites. Following transfection of LNCaP cells at 75% confluency with flag-tagged RANKL and neo plasmid using Lipofectamine 2000 (Invitrogen) in a 6-well plate for 48 h, cells were trypsinized and seeded onto a 15 cm dish. The stable LNCaP-neo and LNCaP-RANKL cells were subjected to G418 selection (400 µg/ml) and single cell clones were isolated and maintained in 200 µg/ml G418 for RNA and protein extraction and immunoassay using 8-well chambered slides (Thermo Scientific, Rochester, NY). Three LNCaP-RANKL and 2 LNCaP-neo cell clones were selected. Since their biochemical phenotypes were similar we used one clone of each for the study.

### Reverse transcriptase (RT) PCR

Total RNA from cells was isolated using RNeasy Mini Kit (Qiagen Sciences, Inc.; Germantown, MD) according to the manufacturer's instructions. Complementary DNA (cDNA) was generated from 3 µg of total RNA using SuperScript® III First-Strand Synthesis System (Invitrogen; 1 µl of cDNA was subjected to PCR analyses using the following primers : RANKL F, 5′-TGG ATC ACA GCA CAT CAG AGC AG-3′; RANKL R, 5′-TGG GGC TCA ATC TAT ATC TCG AAC-3′; E-cadherin F, 5′-GCC AAG CAG CAG TAC ATT CTA CAC G-3′; E-cadherin R, 5′-GCT GTT CTT CAC GTG CTC AAA ATC C-3′; N-cadherin F, 5′-GAT GTT GAG GTA CAG AAT CGT; N-cadherin R, 5′-GGT CGG TCT GGA TGG CGA-3′; vimentin F, 5′-GGA CTC GGT GGA CTT CTC; vimentin R, 5′-CGC ATC TCC TCC TCG TAG-3′, c-Met F, TGGGAATCTGCCTGCGAA; c-Met R, CCAGAGGACGACGCCAAA. The PCR reaction cycles involved an initial denaturation at 94°C for 10 min, 36 cycles of 94°C, 1 min, 55 °C, 30 sec; for RANKL72°C, 1 min followed by a final 72°C for 10 min extension. For E-cadherin and N-cadherin gene amplification, the PCR reactions ran for 32 cycles and the annealing temperatures were 55°C and 47°C, respectively for 30 sec. For vimentin and GAPDH amplification, the PCR reactions ran for 28 cycles with annealing temperatures at 48°C for 30 sec. The amplified PCR products were detected and analyzed on 1% agarose gel.

### Western blot analysis

Cells were lysed in RIPA buffer containing 1× protease inhibitor cocktail (Thermo Fisher Scientific; Rockford, IL) and centrifuged, and the supernatants were collected and quantified using the Bradford Protein Assay (Thermo Fisher Scientific). The cell lysates (20–30 µg) were resolved on a 4–12% Bis-Tris gradient SDS-PAGE (Invitrogen; Carlsbad, CA) under reducing conditions, followed by transblotting onto nitrocellulose membrane (BioRad Laboratories; Hercules, CA), and blocked in 5% non-fat milk/PBST for one hour at room temperature (RT) and incubated with diluted primary Abs in blocking buffer at 4°C overnight. The source and dilution of primary Abs were: RANKL (sc-74261;1∶400), E-cadherin (sc-7870; 1∶1000), vimentin (sc-6260; 1∶500), c-Met (sc-10; 1∶500) (Santa Cruz Biotechnology; Santa Cruz, CA), N-cadherin (#610920; 1∶200) (BD Transduction Laboratories; San Jose, CA and Santa Cruz Biotechnology), p-Met (Tyr-1230/34/35) (#44888G; 1∶500) (Invitrogen), p-NF-κB p65 (Ser536) (#3033; 1∶1000), NF-κB p65 (#4764;1∶1000) (Cell Signaling Technology; Danvers, MA). Production and usage of androgen receptor antibody (PG21;1∶500) were previously reported [24]. The membranes were rinsed 3× with PBST, incubated with peroxidase-conjugated anti-mouse or anti-rabbit secondary Abs at RT for one hour and rinsed 3× with PBST. The signals were visualized using ECL Plus reagent (GE Healthcare Biosciences; Pittsburg, PA). Restore Plus Western Blot Stripping Buffer (Thermo Fisher Scientific) was used prior to re-probing with different Abs.

### In vivo experiments

All animal procedures were performed according to an approved protocol from the Institutional Animal Care and Use Committee (IACUC#2999, Cedars-Sinai Medical Center, and A10-0100, University of British Columbia). LNCaP-RANKL and LNCaP-neo cells (1×10^6^ cells/50 µl PBS) were inoculated intracardially into 5- to 7-week-old male athymic nude mice (Charles River; n = 20 for LNCaP-RANKL and n = 15 for LNCaP-neo). All mice were regularly monitored for metastatic tumor formation which first occurred at 8 wks. Animals were sacrificed at 12 wks.

### Prostate cancer tissue specimens

Formalin-fixed and paraffin-embedded (FFPE) human prostate cancer specimens were obtained from Department of Pathology of the Cedars-Sinai Medical Center (IRB# Pro 00021228). Additional clinical prostate cancer tissue specimens were obtained from Dr. Fouad Habib at the University of Edinburgh, Scotland, and Dr. Hua Yang at the Jilin University, China. Usage of clinical specimens was approved by the human tissue institutional review committees at the respective institutions.

### CRPC xenografts

To establish working protocols for single and sequential multiple QD labeling, we chose to use a LTL-313 CRPC xenograft model since it mimics castration-resistant prostate cancer and provides consistent and readily available multiple tissue specimens critical for the development and establishment of a new MQDL protocol. FFPE tissues from a castration resistant prostate cancer (CRPC) LTL-313 (Living Tumor Laboratory, www.livingtumorcentre.com) xenograft model were obtained from Vancouver Cancer Center, BC Cancer Agency, Vancouver, Canada. The LTL-313 tumor line was developed from prostate needle biopsy specimen from an 80-year old patient diagnosed with Gleason Score 8 prostate cancer with a serum PSA level of 17 ng/ml at the time of diagnosis [25]. The tumor line development protocol was approved by the Clinical Research Ethics Board of the University of British Columbia, in accordance with the Laboratory Animal Guidelines of the Institute of Experimental Animal Sciences (Human specimen use was with approvals from University of British Columbia, Canada (IRB# UBC BCCA REB #H04-60131). Briefly, to establish castration-resistant prostate cancer, fresh LTL-313 tumor tissues from the 5th generation of grafting were cut into 3×3×1 mm pieces and re-grafted into the subrenal capsules of male NOD-SCID mice. The animals were maintained for tumor formation for two months before castration to remove androgens. Three weeks after castration, when the tumor volume was significantly reduced, the remaining tumor tissues were harvested and prepared as FFPE tissue blocks. Xenongraft tissues used in this study were derived from 9 tumors from castrated mice and 5 tumors from 5 intact mouse hosts supplemented with testosterone that had undergone sham operation.

### Immunoassay reagents

The primary antibodies (Abs) and their sources were: mouse monoclonal Abs to human EpCAM (VU-1D9) and RANKL (12A668) from Novus Biologicals (St. Charles, MO); mouse monoclonal Ab to c-Met (25H2) and rabbit monoclonal Ab to E-cadherin (24E10) from Cell Signaling Technology; rabbit polyclonal Ab to androgen receptor, AR, (PG 21), provided by Dr. Gail Prins from University of Illinois (Urbana, IL); goat polyclonal Ab to neuropilin-1 (C-19), rabbit polyclonal Abs to N-cadherin (H-63), Mcl-1 (S-19), p-NF-κB p65 (Ser 536), and VEGF (A-20) from Santa Cruz Biotechnology, Inc.; and rabbit polyclonal Ab to p-c-Met (pYpYpY1230/1234/1235) from Invitrogen. Secondary Abs used in the study were prepared in a cocktail of biotinylated Abs to mouse, rabbit, and goat IgG (Vector Laboratories, Inc., Burlingame, CA). Phosphate-buffered saline (PBS) and streptavidin-conjugated QDs at 565-, 585-, 605-, 625-, 655- and 705 nm wavelengths as 1 µM stock solution were purchased from Invitrogen.

### Immunohistochemical (IHC) staining

IHC followed our published protocol [26] with minor modifications. FFPE sections (4 µm) were deparaffinized, rehydrated, and subjected to antigen retrieval. After incubating in Dual Endogenous Enzyme Block solution (DEEB, Dako, Carpinteria, CA) for 10 minutes, the section was treated with primary antibody diluted with Antibody Diluent solution (Dako) as follows: RANKL(1∶100), c-Met(1∶50), neuropilin 1 (1∶100), androgen receptor (1∶200), N-Cadherin (1∶100), Mcl-1 (1∶100); p-NF-κB p65 (Ser536) (1∶100), VEGF (1∶50) and p-c-Met [pYpYpY1230/1234/1235] (1∶100), vimentin (1∶100), and E-cadherin (1∶200) at 4°C overnight. The section was then washed 3 times in PBST (PBS containing 0.2% Tween 20) for 5 minutes per washing. To detect specific staining, the section was treated for 30 minutes with EnVision^+^ Dual Link System-HRP (Dako), which contained horse radish peroxidase conjugated goat antibodies to mouse and rabbit Ig. The section was washed 3 times for 5 minutes each, and specific stains were developed with 3,3′-diaminobenzidine (Dako).

### Single QD Labeling (SQDL)

The IHC staining protocol was modified for single QD labeling. Streptavidin-conjugated QDs (565-, 585-, 605-, 625-, 655- and 705 nm) were prepared as 10 nM in PBS with 6% IgG-free, protease-free BSA (Jackson Immunoresearch; West Grove, PA). Antigen retrieved tissues or fixed cell samples were incubated in PBS containing 2.5% horse serum (Vector Laboratories) and 20% Streptavidin Block Reagent (Invitrogen) for 20 minutes, followed by treatment with primary Abs as described above in the above immunoreagents section in PBS plus 1% horse serum and 20% Biotin Block Reagent (Invitrogen) at 4°C overnight. After a 3×5 min PBST (PBS+ 0.4% Triton X-100) rinse specimens were incubated with the secondary Ab cocktail at room temperature for 30 min and then incubated with the respective streptavidin-QD at 37°C for 60 min. At the end of each incubation, the specimens were subjected to routine 3×5 min PBST (PBS+ 0.4% Triton X-100) rinse. After the final rinse, the slides were mounted in aqueous mounting media containing 4′6-diamidino-2-phenylindole (DAPI) (Vector Laboratories) for imaging. The Ab dilutions were described in the IHC section above.

### Multiplexed QD Labeling (MQDL)

The single QD labeling protocol was modified for MQDL, in which a single tissue section was subjected to staining for multiple markers in a sequential manner. For each biomarker test, labeling started with a streptavidin blocking, followed by primary Ab reaction and biotinylated secondary Ab incubation, and reaction with streptavidin-conjugated QD at a specified wavelength. Sections were incubated sequentially (with a 3×5 min PBST rinse after each incubation). Primary Abs and dilutions in MQDL were identical to those used for SQDL. The immunoreaction sequences were: 1) Anti-Neuropilin-1 Ab, 2 hours, room temperature; biotinylated horse anti-goat IgG, at room temperature, 30 min; streptavidin-QD705, at 37°C, 1 hour. 2) Anti- p-c-Met Ab, at 4°C, overnight; biotinylated horse anti-rabbit IgG, at room temperature, 30 min; streptavidin-QD655 at 37°C, 1 hour. 3) Anti-VEGF Ab, at room temperature, 2 hours; biotinylated horse anti-rabbit IgG, at roomtemperature, 30 min; streptavidin-QD625 at 37°C, 1 hour. 4) Anti- p-p65-NFκB Ab at 4°C, overnight; biotinylated horse anti-rabbit IgG at room temperature, 30 min; streptavidin-QD565 at 37°C, 1 hour. 5) Anti- RANKL Ab at room temperature, 2 hours; biotinylated horse anti-mouse IgG at room temperature, 30 min; streptavidin-QD585 at 37°C, 1 hour. 6) Mounting in aqueous mounting media containing DAPI. Ab concentrations were described in the IHC section. Sections of FFPE LNCaP-RANKL human prostate cancer cells were used as a positive control. For negative control, primary Abs were replaced with isotype- and species-matched control Abs and applied to an immediately adjacent tissue section, and MQDL was performed in parallel with the tissue slide labeled with the testing primary Abs.

### Image Acquisition

A CRi spectral imaging system (Caliper Life Sciences, Hopkinton, MA) with built-in Nuance v3.1 software was used to document multispectral images following the manufacturer's recommended protocol. For each field of cancer tissue, serial images were acquired at a 10 nm wavelength interval from 450 to 800 nm, a range chosen corresponding to the active fluorescent QDs. It generates an unprocessed image “cube” as a stack of 36 separate images with each image containing the complete spectral information for every pixel at that given wavelength. All images were acquired at 400× magnification, with a 50 milliseconds exposure. To avoid variations in labeling due to cell heterogeneity, five images from different cancerous tissue sites were taken for each tissue specimen for subsequent quantification.

### Image Deconvolution

Image deconvolution or unmixing protocol was used to extract specific labeling by a specified QD. A spectral library for 565, 585, 605, 625, 655, and 705 nm was built for deconvolution by performimg multiple SQDLs using androgen receptor (AR) antibody without final DAPI counterstaining on serially adjacent tissue sections. The positive labeling of AR was confirmed by parallel IHC staining on adjacent tissues. The spectrum of DAPI was obtained by mounting the adjacent tissue in the aqueous mounting medium containing DAPI. Spectra of tissue autofluorescence were acquired for each tissue from a negative control slide (see MQDL section) prepared by IHC without fluorescent markers. Autofluorescence reduction was performed using Real Component Analysis plug-in software. The spectral library was then used to unmix the cube. The separate spectral contributions to the data ‘cube’ are outputted as designated colored intensity maps. These images represent the distribution of each of the QDs and autofluorescence in the tissue. After the deconvolution of the images, the background labeling was filtered and only the true positive signals were shown on the images of the Figures presented.

### Signal Quantification

The spectral library was used to deconvolute the imaging cube to extract labeling of individual QD using inForm v1.3 software (Caliper) following the recommended strategy. First, a training set comprising two classes of tissue was created: ‘cancer’ and ‘non-cancer’. The software was trained on these areas using the spectra of both the DAPI counterstain and the multiplexed QD immunolabeling and tested on 50 randomly selected cells from each image to determine the accuracy of differentiating the two classes. This process was repeated until further iterations no longer improved the accuracy. Histological H/E images were then used to localize cancer cells based on nuclear DAPI labeling. A built-in algorithm was then used to define the cytoplasmic vs. nuclear subcellular regions. Based on an analysis of images at 400× magnification, the optimal threshold settings approximated: fixed scale 200, minimum blob size 20, maximum blob size 10,000, circularity threshold 0, edge sharpness 0, fill hole enabled (nuclear parameters); inner distance to nucleus 1, outer distance to nucleus 7, minimum cytoplasm sample size 1, minimum signal range 0, maximum signal range 65535 (cytoplasmic parameters). To eliminate non-specific signals at the specified spectrum, an acellular area within the field being analyzed was used as background labeling. QD fluorescence intensity in each cell was exported to an Excel spreadsheet (Microsoft, Seattle, WA) and subjected to statistical analysis [27], [28].

## Results

### Development of a quantitative QD labeling protocol for the assessment of gene expression associated with the activation of c-Met signaling and EMT in cultured human LNCaP cells stably tansfected with RANKL

LNCaP bone and soft tissue metastases model: A novel LNCaP bone metastasis model was developed by stable transfection of this cell line with RANKL (LNCaP-RANKL), which drives EMT in the transfected cells. When RANKL overexpressing LNCaP cells were injected intracardially in mice they exhibited an increased incidence of bone and soft tissue metastases (Table 1).

**Table 1:** RANKL overexpressing LNCaP cells induced high incidences of bone and soft tissue metastases.

	Lymph nodes	Bones*	Adrenal glands	Lung
LNCaP-neo	0/15	0/15	0/15	0/15
LNCaP-RANKL	18/20 (90%)	20/20 (100%)	17/20 (85%)	8/20 (40%)

LNCaP-RANKL and LNCaP-neo cells (1×10^6^ cells/50 µl PBS) were inoculated intracardially into 5- to 7-week-old male athymic nude mice (LNCaP-RANKL n = 20; LNCaP-neo n = 15). Metastatic lesions were observed between 2–3 months after injection.

*Micro-CT tomography detected bone lesions in limbs, ribs, jaw, and skull determined by X-ray radiography and confirmed by histopathologic staining (H&E).

**Figure 1:**
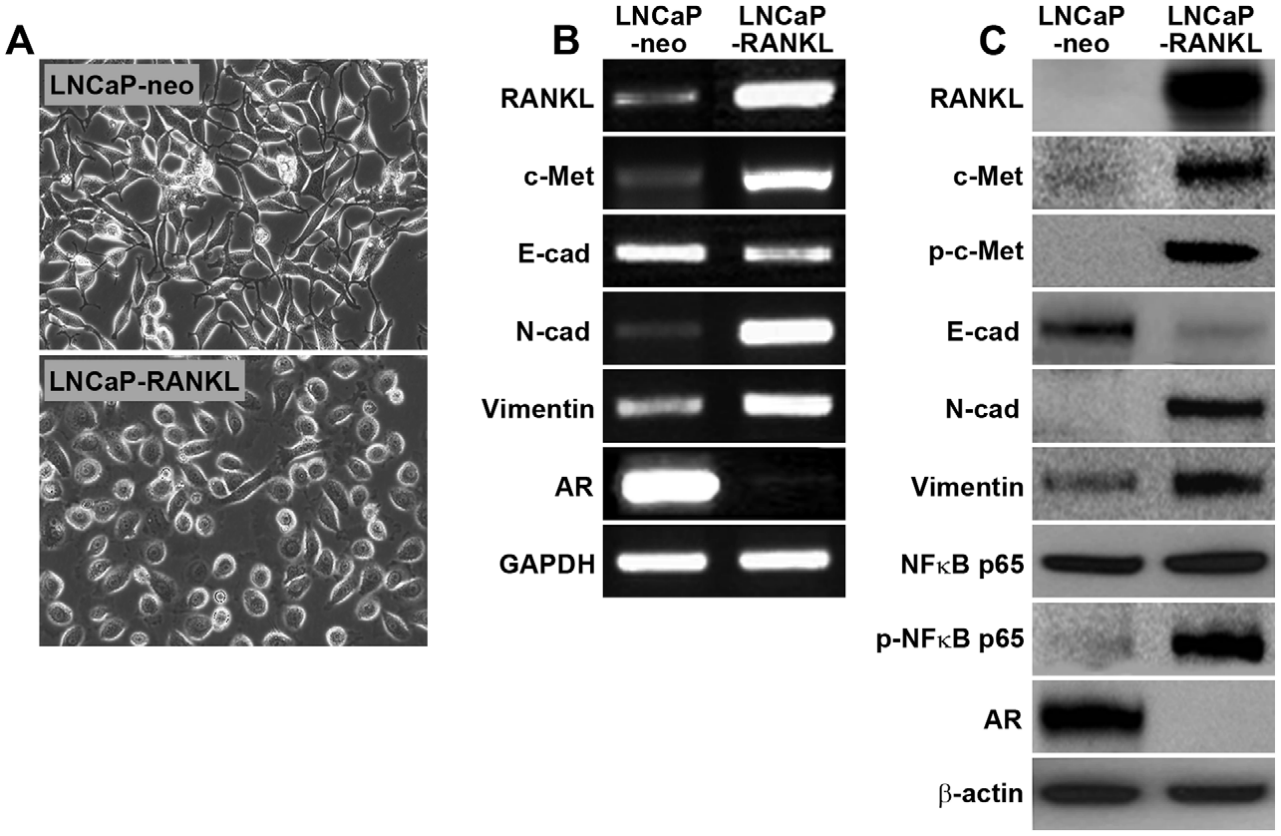
RANKL overexpression induced epithelial to mesencymal transition. RANKL-transfected LNCaP cells induced EMT in histomorphology (A) and gene expression in mRNA (B) and protein (C). Data represent one of 3 RANKL-stably transfected and 2 neo-stably transfected LNCaP cell clones.

**Figure 2:**
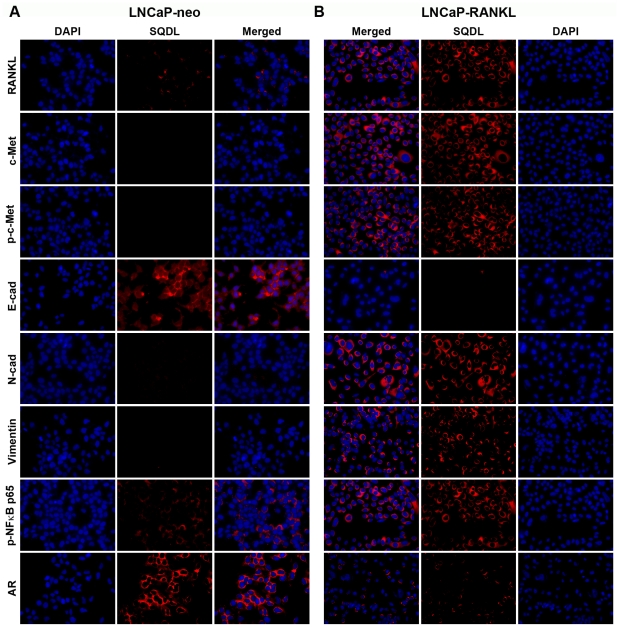
RANKL overexpression activated c-Met signaling components detected by single quantum dot labeling, SQDL. Differential QD labeling of proteins was performed in LNCaP-neo (**A**) and LNCaP-RANKL (**B**) cells using DAPI nuclear staining as reference. For each analysis, QD labeled protein expression is presented in pseudocolor, with the overlaid image of DAPI and QD signals (Merged). ×400.

**Figure 3:**
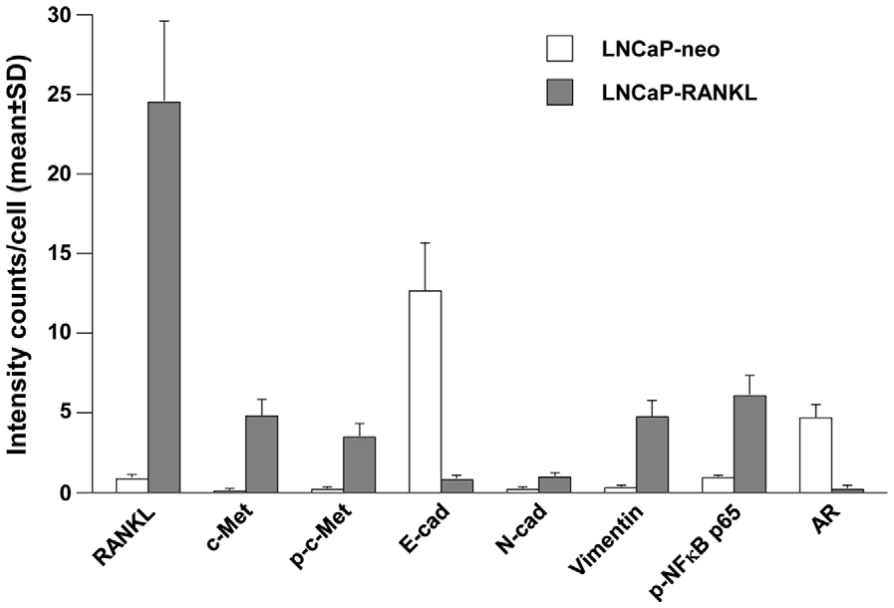
Quantification of differential gene expression subsequent to RANKL overexpression in LNCaP cells. Cell-based average intensity counts from 1,000 each of neo- and RANKL-transfected LNCaP clones were quantified using inForm software. Statistically significant changes in protein expression were observed (*P*<0.05).

Validation of activated c-Met signaling components that lead to EMT by RT-PCR, Western blot and Single Quantum Dot Labeling, SQDL: Comparison of gene expression between stable LNCaP-neo control and LNCaP-RANKL cells using RT-PCR and Western blot showed evidence of activated c-Met signaling through increased expression of c-Met, p-c-Met, and p-NFκB p65 (Figure 1). The cells underwent morphologic (Panel A) and biochemical (Panels B and C) epithelial to mesenchymal transition, with decreased intercellular adhesion mediated by E-cadherin and EpCAM, and increased N-cadherin, vimentin and RANKL. These assessments of c-Met signaling activation and EMT were subjected to SQDL analyses with a specific effort to confirm if c-Met activation and EMT occurred in this cell model of prostate cancer progression. SQDL confirmed that in comparison to control LNCaP-neo cells (Figure 2A), LNCaP-RANKL cells (Figure 2B) had activated c-Met signaling as revealed by an elevated expression of RANKL, c-Met, p-c-Met and p-NFκB p65 and evidence of EMT, a cadherin switch of elevated expression of N-cadherin, vimentin and RANKL but decreased expression of E-cadherin and AR. Quantitative analyses (Figure 3) of 1,000 randomly selected cancer cells by inForm software (Caliper) supported the imaging data in which both AR and E-cadherin expression was drastically decreased and activated c-Met signaling components led to EMT, such as elevated expression of c-Met, p-c-Met, p-NFκB p65, N-cadherin, vimentin, and RANKL were observed in LNCaP-RANKL, when compared to LNCaP-neo cells. This quantitative SQDL protocol was adopted for gene expression analyses of an established CRPC LTL-313 xenograft model.

### Validation of c-Met signaling activation and EMT in the CRPC LTL-313 model with results confirmed by IHC and SQDL

**Figure 4:**
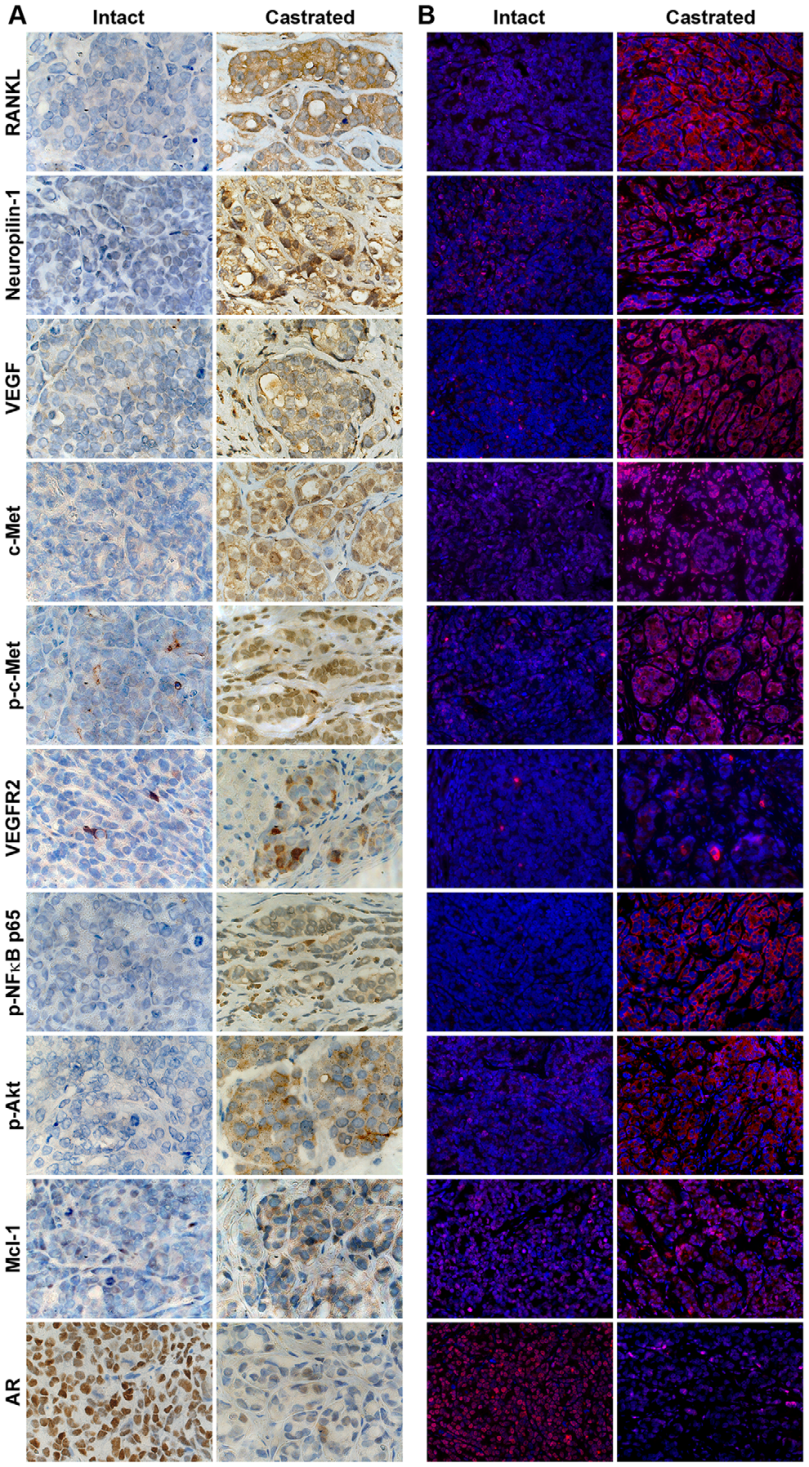
Castration induced c-Met activated and EMT associated proteins. The c-Met activated and EMT associated proteins were detected in the CRPC LTL-313 xenograft model by conventional IHC (A) and SQDL (B). Castration increased a panel of genes in LTL-313 xenograft prostate cancer and decreased AR expression compared to xenografts obtained from intact hosts by both IHC and SQDL. ×400.

To understand the clinical significance of c-Met signaling activation and EMT in the LNCaP-RANKL model and the associated increases in prostate cancer metastases, we defined the c-Met signaling pathway and EMT in a CRPC LTL-313 xenograft model obtained from the Living Tumor Laboratory (livingtumorcentre.com). We included additional c-Met signaling associated genes such as neuropilin-1, VEGF, p-Akt, VEGFR2, Mcl-1 and AR, which were characterized as mediators of c-Met downstream survival signaling [15], [21], [29], [30]. Figure 4 shows that increased c-Met signaling-associated genes and decreased AR expression in CRPC LTL-313 xenografts maintained in castrated male hosts, as assessed by IHC (Panel A) and SQDL (Panel B) compared to the xenografts maintained in intact mice with androgen supplementation.

**Figure 5:**
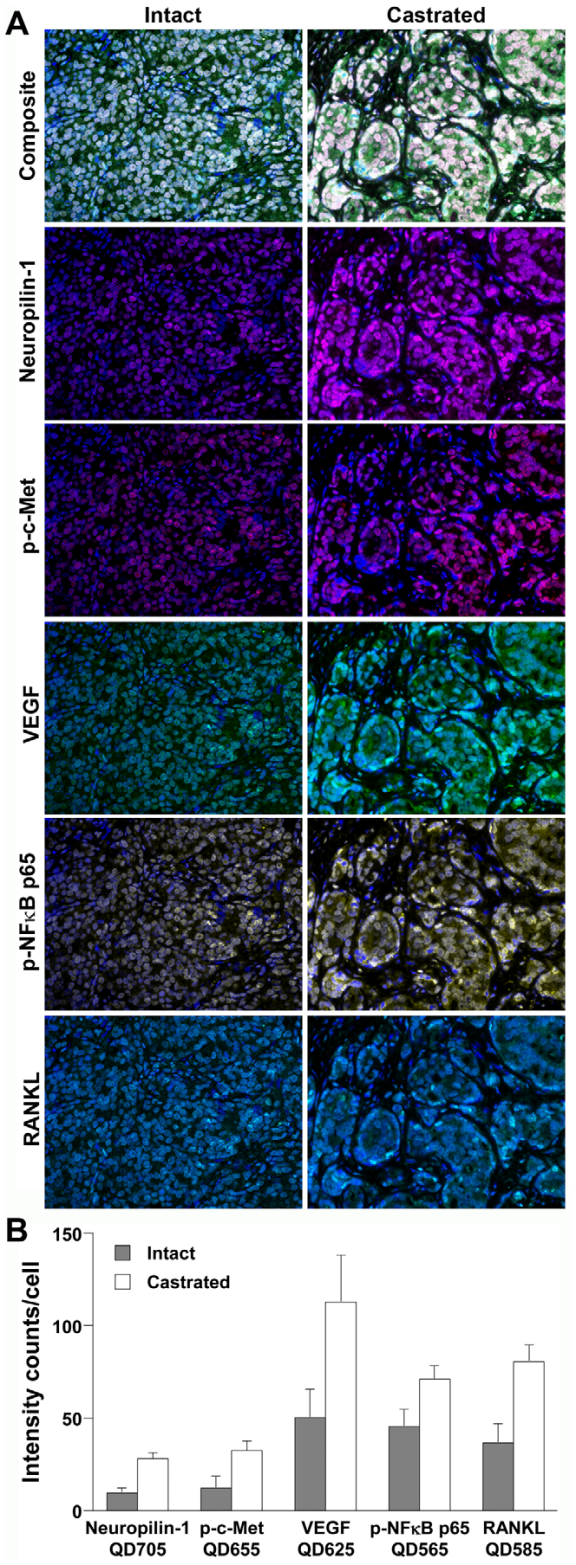
Multiplexed quantum dot labeling (MQDL) simultaneously detected c-Met activated and EMT genes in a CRPC LTL-313 xenograft model. Single tumor tissue sections from intact and castrated hosts were subjected to sequential MQDL. Enhanced expression of c-Met activated genes was detected in tumor tissues from castrated hosts. (**A**) A stack of multiple images (Cube), nuclear staining (DAPI), and individual gene expression (in pseudocolors) are shown. Gene expression signals were superimposed with DAPI nuclear signals to yield a composite image for analysis. ×400. (**B**) Significant differences in gene expression were found by quantification analysis of 1,000 cells in each tumor specimen using inForm software (*P*<0.05).

**Figure 6:**
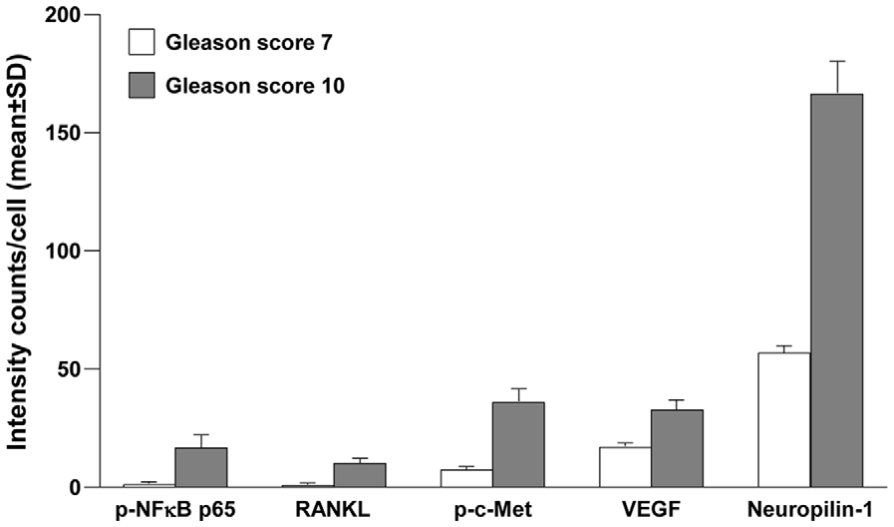
MQDL assesses differential gene expression in clinical primary prostate cancer specimens. C-Met-activated and EMT protein expressions in 5,000 cells of each representative prostate cancer tissues of Gleason scores 7 (3+4) and 10 (5+5) were compared. Expression intensities were quantified with the inForm software and statistically compared (*P*<0.05).

MQDL analysis demonstrated activated c-Met and EMT in a CRPC LTL-313 xenograft model and primary and metastatic human prostate cancer tissue specimens. A series of human prostate cancer xenografts obtained from the Living Tumor Laboratory was used to test the quantitative MQDL protocol. We selected the LTL-313 model for this task because this model exhibits CRPC characteristics with a propensity for primarily lymph node, lung and liver metastases with infrequent bone metastasis, when the tumors are implanted and grown under the renal capsules. We tested the hypothesis that tumors grown in castrated mice would develop castration resistance and have activated c-Met signaling leading to EMT, when compared to tumors grown in mice supplemented with exogenous androgen (Figure 5A). Three approaches were taken: 1) to establish a robust MQDL protocol to detect and quantify a panel of gene expressions on the CRPC LTL-313 model tissue specimen with results compared with SQDL; 2) to use this established quantitative MQDL protocol to confirm if activation of c-Met and EMT occur in the CRPC LTL-313 tissues maintained in the castrated hosts; and 3) to demonstrate by the standardized MQDL protocol the activation of c-Met and EMT induction in both primary and skeletal metastatic human prostate cancer tissues. Figure 5B shows the quantitative MQDL analyses of neuropili-1, p-c-Met, VEGF, p-NFκB p65, and RANKL, previously reported to be associated with c-Met signal activation [15], [22] and EMT [26], [31] in human prostate cancer cells and in the CRPC LTL-313 model. We showed activated c-Met signaling and EMT, as exhibited by increased expression of neuropilin-1, VEGF, and RANKL protein and activation of p-c-Met and p-NFκB p65 in the CRPC LTL-313 tumor model with higher expression and activation of these genes in LTL-313 tumor xenografts maintained in castrated compared to intact mice. As an internal control for minimizing the possible fluorescence interference of multiple QD probes, we performed MQDL side by side with the SQDL protocol (see above). The elevated expression of the 5 studied genes was found to be statistically significant by inFom quantitative analyses (Figure 5B). We determined this panel of 5 genes in primary prostate cancer specimens with different Gleason scores and found that expression of neuropilin-1, VEGF, p-c-Met, RANKL, and p-NFκB p65 were more elevated in poorly differentiated (Gleason score 10) than moderately differentiated (Gleason score 7, 3+4) prostate cancer (Figure 6). These results, taken together, confirmed that QD labeling is highly sensitive and versatile and can be used for multiplexing gene expression profiles in experimental models at the single cell level.

**Figure 7:**
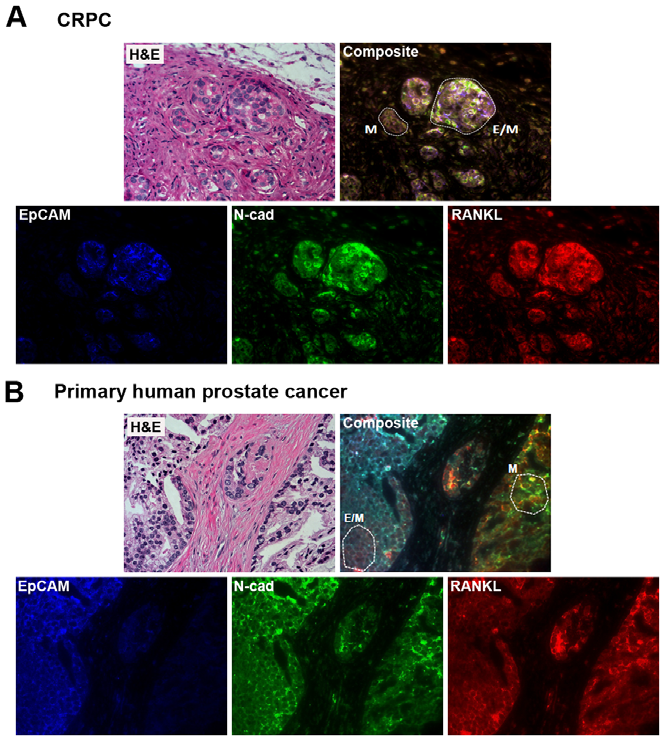
MQDL detects EMT biomarkers in CRPC LTL-313 prostate tumors harvested from castrated hosts and from clinical prostate cancer. (**A**) The CRPC LTL-313 model showed androgen deprivation induced more abundant expression of EMT markers in a cluster of cells within the tumor region, as revealed by QD labeling of EpCAM, N-Cad, and RANKL. (**B**) Similar EMT biomarkers were detected in a clinical primary prostate cancer specimen (Gleason score 6; 3+3) with documented bone metastasis. M denotes cells that completed EMT and E/M indicates cells undergoing partial EMT. ×400.

**Figure 8:**
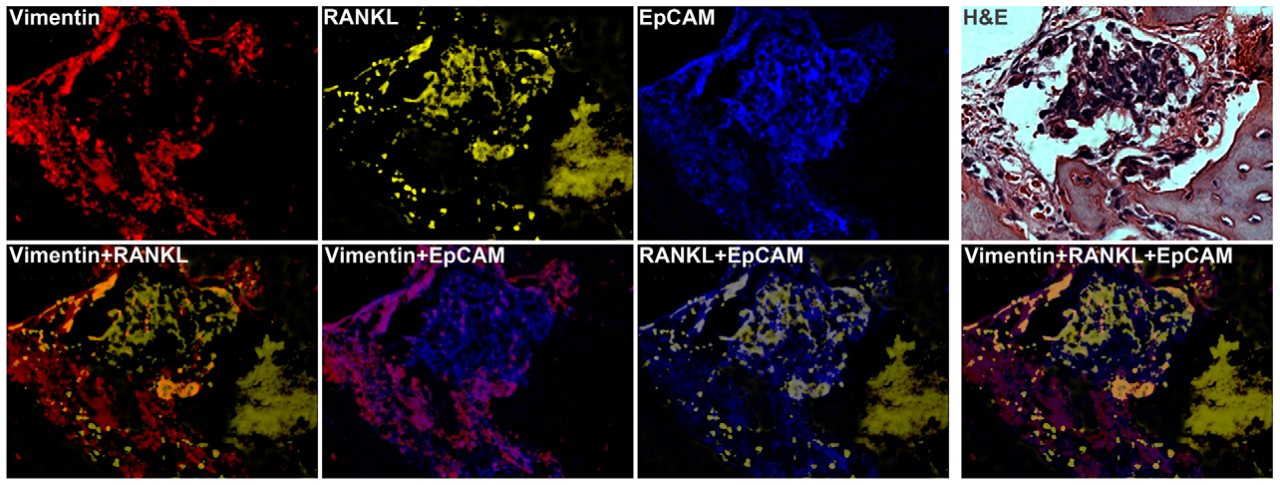
MQDL detects EMT biomarkers in clinical bone tissue specimens. A representative specimen of human prostate cancer bone metastasis co-expressed high levels of epithelial EpCAM, and mesenchymal RANKL and vimentin proteins. ×400.

Furthermore, we showed that some clusters of LTL-313 tumor cells grown as xenografts in castrated male hosts underwent EMT (marked as M in Figure 7A, Composite panel) and others only partially transitioned to express both E and M markers (marked as E/M in Figure 7A, Composite panel). Decreased expression of EpCAM, an epithelial biomarker, with concomitant increased expression of mesenchymal markers, such as N-cadherin and RANKL, was observed (Figure 7A, Deconvoluted panels). These data support the conclusion that castration promotes EMT in prostate cancer cells *in situ*. Similar EMT was detected by MQDL in clinical primary prostate cancers (Figure 7B). A cluster of prostate cancer cells underwent EMT (M) while the other clusters of cells underwent partial EMT (E/M) (Figure 7B, Composite panel). Furthermore, Figure 8 demonstrates the co-expression of epithelial EpCAM and mesenchymal RANKL, and vimentin biomarkers in a metastatic bone tissue specimen.

## Discussion

Understanding the underlying molecular signaling pathway in CRPC and prostate cancer bone and soft tissue metastases could benefit patient care through improved disease surveillance and selection of patients for more effective targeting. Considering the limited amount of tissues and cells available from biopsies and needle aspirates, and the challenges of tumor cell heterogeneity, MQDL offers a valuable multiplexing capability to analyze changes in cell signaling network components, at the single cell level during prostate cancer progression in both preclinical models and clinical specimens. The advantages of quantitative MQDL over conventional IHC are: 1) Data obtained by quantitative MQDL could contain spatial and temporal information on the tissue specimens at the single cell level that can be easily integrated into the already-known histomorphometric and immnohistochemical information obtained from the tumor tissues or cells. 2) The cell signaling network defined in tissue specimens could have predictive value for assessing the progression and therapeutic responsiveness of the prostate cancer. 3) Quantitative MQDL is highly efficient for tissue and cell utilization and can be further developed to monitor simultaneously multiple cell signaling networks in human cancer cells *in situ*. The results of our studies using quantitative MQDL suggest that activation of c-Met signaling is responsible for driving prostate cancer cells to undergo EMT, with prostate cancer cells acquiring increased capability for cell migration, invasion and metastasis. The quantitative aspect of the MQDL protocol is supported by a preclinical animal model in which LNCaP-RANKL cells, shown to overexpress RANKL, had activated c-Met signaling (Table 1, Figures 1,2,3), and underwent EMT by acquiring increased ability to metastasize to mouse skeleton and soft tissues, compared to parental LNCaP-neo cells. In parallel, we presented evidence of activated c-Met and EMT in a preclinical CRPC LTL-313 tumor xenograft model (Figures 4, 5, and 7A). Using the established and validated MQDL protocol, we consistently observed activated c-Met with evidence of EMT in poorly differentiated (high Gleason score) and bone metastatic clinical prostate cancer specimens (Figures 6 and 8) [15], [26], [32]. Taken together, we suggest that activation of c-Met signaling and subsequent induction of EMT is probably a common feature of prostate cancer cells, xenograft models and clinical prostate cancer specimens exhibiting an increased propensity for lethal progression. These results suggest the possible link between c-Met-mediated signaling activation, EMT and prostate cancer aggressiveness developed in a castration-resistant state. This increased c-Met signaling network in prostate cancer cells could be considered as one of the reasons why targeting c-Met and VEGFR2 kinases with a small molecule, cabozantinib (XL-184), resolved bone and soft tissue metastases in a broad spectrum of solid tumors in patients [20], [21]. Because of the small sample size used for the determination of c-Met signal activation in clinical prostate cancer specimens, a large scale of evaluation using clinical prostate cancer specimens with known status of c-Met activation (e.g. sensitive vs. resistant to cabozantinib) is warranted.

Several technical issues with MQDL deserve special attention. 1) Conjugation non-covalently of streptavidin-coated QD to biotinylated secondary whole Ab is preferred to the direct conjugation of QD to secondary Ab fragments via reduced sulfhydryl-amine coupling. The former non-covalent method of QD detection through the biotin-avidin complex in our practice provides high labeling specificity and intensity. 2) Florescence interference and florescence resonance energy transfer can present a challenge for designing a suitable protocol to assess gene expression in cells. Sweeney [5] provided earlier evidence that order of the addition of antibodies and QDs could affect cell labeling read-outs. We suggest conducting a series of combinational tests to determine the optimal reaction sequence and using matched primary Abs and QDs, a parallel set of negative control Ab IgGs, and when possible confirming the MQDL results with SQDL, IHC and Western blots. 3) The quality of the tissues and cells and non-specific labeling should be carefully determined in the immediately adjacent tissue sections to the actual sections used for data acquisition and analyses. 4) For internal controls, β-actin and EF1-α, two routinely used internal protein loading controls for western blotting, were assessed in our current protocol. We found that β-actin and EF1-α expression levels fluctuated depending on the QD-conjugates used and the levels were different between prostate tissues obtained from intact and castrated hosts in preclinical models. These results are in agreement with the report of Yurube T *et al.* [33] using β-actin as a control gene for immunofluorescence detection of gene expression in a rat tail disc degeneration model and found that β-actin was significantly down-regulated rather than staying unchanged. Before establishing a well-accepted internal control for MQDL, we used stringent negative subtype matched antibodies as controls.

In summary, MQDL is demonstrated to be a highly dynamic technique for assessing multiple gene expression at the single cell level in formalin-fixed cell and tissue specimens. This protocol could be of great value for assessing cell signaling network in tissues and cells obtained from the clinic or from the laboratory. Because of the unique photo-physical properties of QDs, MQDL could provide a sensitive method to determine levels of gene expression relevant to cell signaling networks or special biomarker expression even when clinical materials are limited.
